# Research on Unsafe Behavior of Construction Workers Under the Bidirectional Effect of Formal Rule Awareness and Conformity Mentality

**DOI:** 10.3389/fpsyg.2021.794394

**Published:** 2021-12-15

**Authors:** Zhen Li, Xiaoyu Bao, Yingying Sheng, Yu Xia

**Affiliations:** ^1^School of Management, Jiangsu University, Zhenjiang, China; ^2^Faculty of Business and Economics, The University of Melbourne, Parkville, VIC, Australia

**Keywords:** formal rule awareness, conformity mentality, safety cognition, unsafe behavior, multi-agent modeling

## Abstract

At present, China’s engineering safety management has developed to a certain level, but the number of casualties caused by construction accidents is still increasing in recent years, and the safety problems in the construction industry are still worrying. For purpose of effectively reducing construction workers’ unsafe behavior and improve the efficiency of construction safety management, based on multi-agent modeling, this paper analyzes the influencing factors during construction workers’ cognitive process from the perspective of safety cognition, constructs the interaction and cognition of the agent under the bidirectional effect of formal rule awareness and conformity mentality model, and set behavior rules and parameters through the Net Logo platform for simulation. The results show that: Unsafe behavior of construction workers is related to the failure of cognitive process, and the role of workers’ psychology and consciousness will affect the cognitive process; The higher the level of conformity intention of construction workers, the easier it is to increase the unsafe behavior of the group; Formal rule awareness can play a greater role only when the management standard is at a high level, and can correct the workers’ safety cognition and effectively correct the workers’ unsafe behavior; Under certain construction site environmental risks, the interaction between formal rule awareness and conformity mentality in an appropriate range is conducive to the realization of construction project life cycle management. This study has certain theoretical and practical significance for in-depth understanding of safety cognition and reducing unsafe behavior of construction team.

## Introduction

The construction industry plays an important role in promoting the development of the national economy (Zhou et al., 2015). According to the 2020 National Economic and Social Development Statistical Bulletin of the National Bureau of Statistics (China Statistics, 2021), the total added value of the construction industry throughout the year was 7,299.6 billion yuan, an increase of 3.5% over the previous year. However, the safety production situation in the construction industry is still very serious. According to a report from the Ministry of Housing and Urban-Rural Development of the People’s Republic of China (Standardization of Engineering Construction, 2020), in 2019, a total of 773 housing engineering safety accidents occurred nationwide, and 904 people died, a year-on-year increase of 5.31 and 7.62%. It is worth pondering that at present, China’s engineering safety management has developed to a certain level. The attitude of engineering managers toward safety issues, the rules and regulations of safety management, the application of equipment and technology, and the safety atmosphere and awareness of construction subjects are all showing a good development trend. However, why are engineering safety accidents still emerging one after another?

Research evidence has shown that human unsafe behavior is the most important reason affecting construction safety production (Park et al., 2020; Zhang et al., 2020). Among the accidents on construction sites, 90% are caused by human errors (Newaz et al., 2020), and 88% of construction engineering accidents involve human unsafe behavior (Suraji et al., 2001). An effective strategy to enhance the safety production management and the safety production performance of construction projects is to prevent and control unsafe behaviors of workers (Fang et al., 2019; Jiang et al., 2020).

Workers’ unsafe behavior is a manifestation of cognitive failure. Workers’ safety cognition belongs to the category of psychological research on worker behavior (Wang et al., 2016), which has become one of the most concerned issues in construction site safety research (Liao et al., 2017). Recently, domestic and foreign scholars have carried out a large number of studies on cognitive process (Cheng, 2020), cognitive failure (Cheng, 2020) and cognitive factors (Goh and Sa’Adon, 2015), and analyzed factors such as safety awareness (Fang and Cho, 2016), safety attitude (Han et al., 2019) and behavior control (Hamilton et al., 2019). The impact of this has made an important contribution to clarifying the causal relationship of safety cognition. However, the research on unsafe behaviors from the perspective of construction workers’ individual cognition is still insufficient, ignoring the role of construction workers’ psychological and consciousness changes in the interaction process. Safety cognition is a process of continuous dynamic change, which will be affected by various changing factors, such as workers’ psychological activities and environmental factors. As the most basic and core component of the construction team, construction workers, the interaction of individuals in the group will directly affect the workers’ own safety cognition (Peiró et al., 2020). Conformity mentality and formal rule awareness lead to unsafe behavior through the interaction of construction workers, which is a major problem in team management (Ahn et al., 2013; Aljadeff et al., 2020). Conformity is defined as Individual behavior is affected by the behavior of team members, judged and cognized according to the internal norms of the team, which will force individual behavior to be consistent with group behavior and finally manifested as gregariousness (Wang and Chen, 2021), and affects the agents’ cognitive differences and risk steady-state cycle (Liu and Ding, 2021). Workers’ formal rule awareness determines the weight of formal standards relative to perceived standards (Ahn et al., 2013). When an individual forms an internal standard and there is a difference between the internal standard and the formal standard, the workers’ awareness of formal rules often adjusts themselves to reduce the difference to accept the behavior (Carver and Scheier, 1982). It determines the degree of workers’ recognition of the rules and regulations formulated by the management, and affects workers’ learning, recognition and compliance with management norms. It is an important research content of safety production management. Construction workers have been engaged in production and life in teams and groups for a long time. The mutual influence and interaction between agent and the environment and between agents have provided impetus for system evolution (Zhang et al., 2003). On the one hand, formal rule awareness and the conformity mentality affect the binding force of management norms. On the other hand, the colleague effect establishes the behavioral connection between individuals and colleagues and influences each other. As a result, workers will not only adjust their own decisions based on colleagues’ behavioral decisions, but may also have different perceptions of production tasks and risk identification. Considering the two-way effect of conformity mentality and formal rule awareness can clarify the decision-making basis and cognitive differences of unsafe behaviors of construction workers, ensure that management norms play a restrictive role and achieve the objectives of safe production management.

Although more and more scholars begin to pay attention to the role of construction workers’ cognition and psychology, how to use workers’ individual safety cognition to reduce the unsafe behavior of construction team and obtain management enlightenment can be further studied. Therefore, based on the analysis of the influencing factors of workers’ safety cognition, this study explores the relationship between safety cognition and unsafe behavior, establishes the relationship between workers’ conformity effect and rule consciousness and workers’ cognitive process, and verifies the importance of correct workers’ cognitive process; Analyze the role of formal rule awareness and conformity mentality in group unsafe behavior and the improvement effect on individual and group behavior from the cognitive process of workers, in order to obtain effective management strategies and prevent unsafe behavior of construction team. This study innovatively considers the influence of the two dimensions of rule awareness and conformity mentality, and studies the effect of them on the unsafe behavior of construction teams under different situation combination; On the other hand, it combines workers’ safety cognitive process with risk perception to study workers’ behavior decision-making, so as to enrich the research on construction workers’ cognitive psychology. At the same time, using computational experiments to simulate, to a certain extent, promote the process of combining psychological theory with computational simulation methods to study practical problems.

In order to study the above problems, this article uses multi-agent modeling (ABM) to simulate the evolution of construction workers’ unsafe behavior decisions. Construction workers are a group of complex and diverse agents, whose heterogeneity leads to the diversity of behavioral decisions. In view of the advantage that ABM can simulate the interaction between complex, non-linear and discrete subjects, it is convenient to observe the group effect produced by individuals from bottom to top (Li et al., 2021). Therefore, multi-agent modeling is the most appropriate. Considering the advantages and disadvantages of each simulation platform, in order to fully describe the characteristics of the agent and environment, combined with the applicable scenarios of relevant software, this paper selects the Net logo platform to simulate and analyze the unsafe behavior of construction workers. In the second part, this paper first combs the general model of construction workers’ interaction and safety cognition, and explains the process and main variables of safety cognition combined with the actual environment of construction workers. Then, we summarize the relationship among some influencing factors of construction workers’ cognitive process, establishes the behavioral rules of workers’ social cognitive mechanism of unsafe behavior, and initializes the model. Finally, the third part carries out experimental simulation to study their two-way effect by the combination of formal rule awareness and conformity mentality.

## Model Development

### Agent Interaction Setting

Based on the question of what is the cognitive process construction workers who adopt dangerous behavior and using multi-agent modeling, this paper aims to study the occurrence mechanism of workers’ unsafe behavior under the two -way action of formal rule awareness and conformity mentality. The environment of the construction site is complex. Due to different management levels and technical complexity, there will be great differences in environmental risks. At present, there is no unified classification standard for the classification of environmental risk on the construction site, but various studies show that the environmental risk on the construction site will affect the unsafe behavior of workers. This model introduces site risk (*SA*) to describe the degree of unsafe construction site. For example, the site risk is 15%, which means that the worker has a 15% probability of being in a dangerous environment. At the same time, workers will get an actual site risk *AR*(actual risk), the actual site risk is defined to obey the Normal distribution*N* (μ, σ^2^).

**FIGURE 1:**
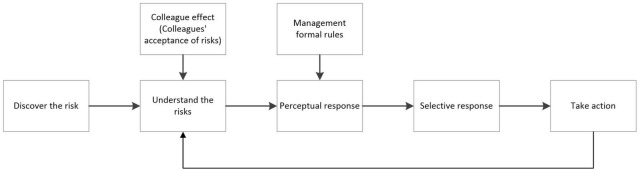
Worker interaction diagram.

In this paper, the interactive behavior of agents is mainly reflected in the learning and imitation behavior among workers. There is an interactive relationship between workers’ behavior and decision-making, and they often deviate from their original choice and make decisions in line with group behavior preferences (Yang et al., 2009). The learning and imitating behaviors of workers in team work is a major way to replicate unsafe behaviors. Research has found that if someone in the construction group takes unsafe behavior, it is likely to trigger herd behavior (He et al., 2020). Workers’ behavior is usually affected in two ways. One comes from the colleague effect, that is, the influence of the behavior of surrounding workers. When making decisions, workers will refer to the actions taken by their colleagues; the other comes from the formal rules set by the management, which can restrict workers’ unsafe behaviors. The interaction between workers, colleagues and management is shown in Figure 1. The colleague effect and the formal rules of management mainly affect the second and third stages of workers’ safety cognition. The production and life circle of construction workers are mainly concentrated within the team, and the interaction between workers is significant. The personal sense of security mainly depends on the safe words and deeds of team members, accident occurrence, etc, (Han et al., 2015). In the construction environment, the most direct impact that workers receive comes from their colleagues. Research shows that co-workers’ behavior is the main factor affecting safety cognition (Qiu and Yu, 2019). When workers perceive risks in the second stage, they are not only derived from their own perception of risks, but also affected by the acceptance of risks by surrounding workers. As a decision maker, the ability of managers and the management decisions they make will have a significant effect on workers’ perception and decision-making and safety performance (Cao et al., 2011). Workers in construction team must consider the formal rules set by the management when they perceive the reaction to form a risk acceptance.

Behavior is the product of cognition (Zhang and Fang, 2012), so cognition has an important influence on the occurrence and evolution of behavior. Workers may have correct perceptions of the real environment, or they may have distorted perceptions and make wrong decisions. In this process, people obtain information, process information, and finally reflect it in the agent’s behavior. Based on the cognitive process proposed by Fang Dongping and the actual environment of construction workers, this article explains the safety cognitive process and main variables as follows. The cognitive process of workers will go through the following five stages:

(1) Discovery of hazard information. It refers to whether workers can find the source of hazards at the construction site, and whether they can find hazards is the first step to avoid unsafe behavior. Whether workers can discover on-site risks depends on the workers’ safety awareness (*SA*) and safety knowledge (*SK*). The higher the safety awareness and the richer the safety knowledge the workers have, the easier it is to identify the field risks.
(2) Understand hazard information. After workers discover the risks on site, they evaluate the risks.
(3) Perceived response. Workers retrieve their own long-term memory, and at the same time, they will be affected by factors such as the external environment to form a risk acceptance (*RA*).
(4) Select response. At this stage, workers will decide which response mode to take from the possible responses.
(5) Take action. This refers to whether workers can implement their own decisions. For example, when workers decide to take safety actions, they need to control themselves, make no mistakes, avoid sudden accidents, and fully implement their decisions.

**FIGURE 2:**
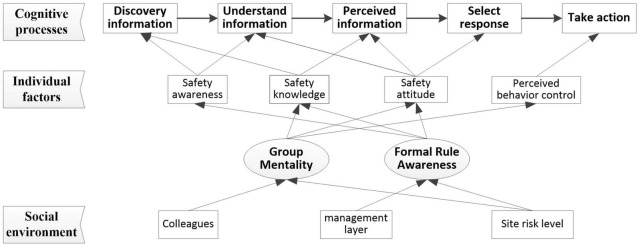
Analysis on influencing factors of construction workers’ cognitive process.

Workers will be affected by multi-dimensional factors when they are in the process of safety cognition, including individual factors and environmental factors (Ye et al., 2020). Individual factors refer to factors such as workers’ own safety awareness, safety knowledge and other factors that affect their cognitive level. Factors such as behavior feedback, demonstration effects, and safety training will affect workers’ individual factors. We define them as external environmental factors. We combed the relationship between some influencing factors of workers’ cognitive process, as summarized in Figure 2. Among them, the three parameters of environmental risk level, conformity mentality and formal rule awareness actually represent what kind of risk environment workers are in, how fast workers evaluate workers’ behavior and regard it as their own code of conduct, and the gap between formal rules and workers’ internal standards. The conformity mentality can be understood as the dependence of construction workers on workers’ behavior. Workers form subjective norms through learning and imitating the behavior of co-workers. Whether workers prefer their own judgment or colleagues’ behavior still has supplementary research space on the impact of this on unsafe behavior. The formal rule awareness of construction workers affects the acceptance of management norms. Management can improve workers’ formal rule awareness through safety training and safety education, which will have an impact on individual factors such as construction workers’ safety attitude, and then affect workers’ safety cognition process.

### Agent Behavior Rules Setting

**FIGURE 3:**
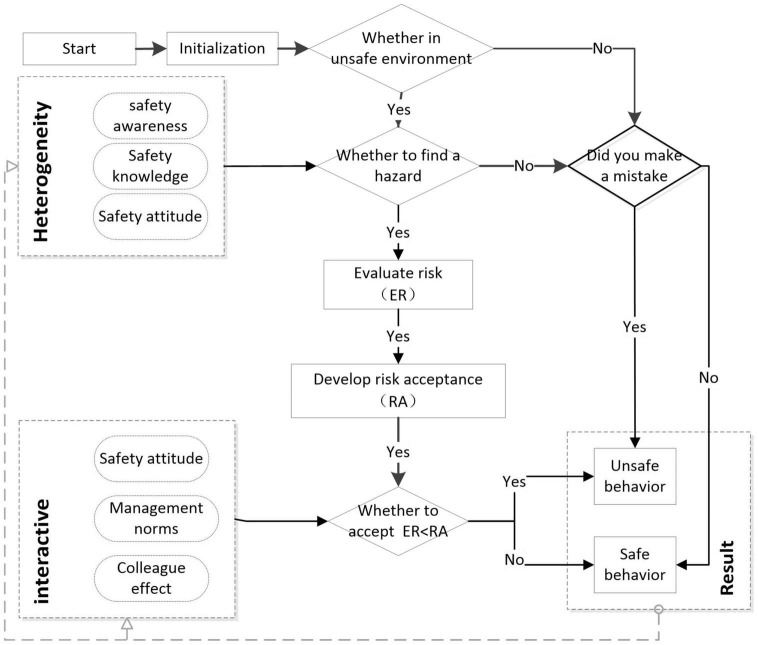
Agent behavior rules.

Based on the cognitive process and risk perception that affect the unsafe behavior of construction workers, we established the behavioral rules of the social cognitive mechanism of unsafe behaviors of workers. Figure 3 summarizes the main rules of the agent’s actions. When a worker is in a construction site with a certain risk, the worker first judges whether the site risk can be found, and then perceives the risk information. Their own safety attitude, colleague effect, and management norms will affect the worker’s risk acceptance. Workers judge whether they accept the risk according to their personal risk acceptance, and finally take safe or unsafe actions.

(1) Discovery of hazard information stage

When construction workers enter the working environment, whether they can find dangerous mainly depends on the workers’ safety knowledge and safety awareness. With less safety knowledge, workers cannot recognize hazards; with lower safety awareness, workers will not pay attention to the dangers around them. Whether workers can find danger at time *t* ($F\,R_{i}^{t}$) is calculated by formula (1).


(1) $$F\,R_{i}^{t}=\left\{ \begin{array}{c} 0,r\,a\,n\,d\,\left(0,1\right)\geq S\,A_{i}^{t}\times S\,K_{i}^{t}\\ 1,r\,a\,n\,d\,\left(0,1\right)<S\,A_{i}^{t}\times S\,K_{i}^{t} \end{array}\right.$$

$S\,A_{i}^{t}$ is safety awareness of worker *i* at time *t*, $S\,K_{i}^{t}$ is safety knowledge of worker *i* at time *t*, $F\,R_{i}^{t}=0$ represents that the worker *i* did not detect and discover the risk or danger at time *t*, $F\,R_{i}^{t}$ = 1 represents the worker *i* discovered dangerous information at time *l*.

(2) Perceived risk stage

If workers find a danger, they will then perceive the risk. Risk perception is workers’ own subjective perception and assessment of the dangerous environment they are in Choi and Lee (2018). Therefore, even in the same site risk environment, the risk perceived by each worker will be different, and this difference can be described by $p_{t}^{i}$ in the model. $p_{t}^{i}$ refers to the construction worker *i*′s risk perception coefficient at the moment of *l*, which reflects the tendency of individuals to overestimate or underestimate the risk. A value greater than 1 indicates that the agent *i*′s perceived risk is greater than the actual risk at time *l*. Workers affected by their own safety attitudes will change from actual risk (AR) to perceived risk (PR). The agent *i*’s perceived risk ($P\,R_{t}^{i}$) at time *l* is calculated as follows.


(2) $$P\,R_{t}^{i}=p_{i}^{t}\,A\,R_{i}^{t}$$

(3) $$p_{i}^{t}=p_{i}^{t-1}-\left(A\,T_{i}^{t}-A\,T_{i}^{t-1}\right)$$

$A\,T_{i}^{t}$ is the agent *i*′s safety attitude at time *l*. If the worker’s safety attitude becomes higher, which means that the worker pursues risk-taking, then the risk perception coefficient will decrease.

(3) Perceptual reaction stage

After the construction workers perceive the risk, they begin to take action. The theory of risk steady state believes that the ability to perceive risks and their acceptability of danger are the two important perspectives for workers to make behavioral decisions (Wilde, 2010). In other words, if the worker’s perceived risk greater than their own risk acceptance threshold, the worker will take safe actions to avoid a safety accident. Considering the research of Choi et al. (Choi and Lee, 2017), in addition to factors derived from the workers themselves, the risk acceptance of workers is also affected by team norms and managerial norms. In equation (4), the worker *i*′s risk acceptance ($R\,A_{i}^{t}$) includes factors such as workers’ safety attitudes, colleague effects, and formal rules.


(4) $$R\,A_{i}^{t}=\left(1-a_{i}\right)\,\left[\left(1-c_{i}\right)\,A\,T_{i}^{t}+c_{i}\,C\,o_{t}^{i}\right]+a_{i}\,W\,N_{i}^{t}+\varepsilon$$

$W\,N_{i}^{t}$ is management norms, ε is the error effect that is not considered in the model, $C\,o_{i}^{t}$ is the colleague effect, which is specifically reflected in the risk acceptance of surrounding colleagues obtained by workers observing the behavior of co-workers, and is defined as the risk acceptance of the 20 nearest co-workers around the worker *i* at time *l*. *a*_*i*_ is workers’ formal rule awareness, *c*_*i*_ is conformity intention, indicates the workers’ willingness to follow the crowd, and refers to the degree to which workers learn and emulate the unsafe behaviors of their surrounding workers.

(4) Selective reaction stage

In this process, workers will compare perceived risks and risk acceptance to decide whether to take safe or unsafe behaviors. When the perceived danger is less than the risk acceptance, it means that the danger perceived by the worker is within his acceptable degree, the worker will think that the risk is not very high and take risks to favor unsafe behavior; When perceived danger exceeds risk acceptance, the worker believes that the situation is beyond their acceptance and will adopt safe behaviors to ensure their own safety, As shown in formula (5).


(5) $$U\,B_{i}^{t}=\left\{ \begin{array}{cc} 0 & P\,R_{i}^{t}>R\,A_{i}^{t}\\ 1 & R\,A_{i}^{t}<P\,R_{i}^{t} \end{array}\right.$$

$U\,B_{i}^{t}$ indicates whether the worker *i* will take unsafe behaviors at time *l*, $U\,B_{i}^{t}=1$ represents the agent *i* will tend to take safe actions at time *l*, $U\,B_{i}^{t}=0$ represents the agent *i* will prefer taking safe actions at time *l*.

When the worker’s perceived risk is within the threshold and the worker has taken unsafe behavior, a safety accident may occur, at this time $A\,c\,c\,i\,d\,e\,n\,t_{i}^{t}=1$. It is also possible that a safety incident did not happen by luck, then $A\,c\,c\,i\,d\,e\,n\,t_{i}^{t}=0$. The probability of an accident is calculated according to formula (6).


(6) $$A\,c\,c\,i\,d\,e\,n\,t_{i}^{t}=\left\{ \begin{array}{cc} 1 & U\,B_{i}^{t}=1,r\,a\,n\,d\,\left(0,1\right)<S\,R\\ 0 & U\,B_{i}^{t}=1,r\,a\,n\,d\,\left(0,1\right)>S\,R,o\,r,U\,B_{i}^{t}=0 \end{array}\right.$$

If a safety accident occurs after a worker has taken an unsafe behavior, the occurrence of the accident will affect the worker’s safety attitude. There is also a situation that even if the worker decides to take a safe behavior, but because of mistakes or unable to control their own behavior, it will also lead to accidents. If workers take unsafe behaviors and accidents occur, workers’ safety attitudes will decrease; if workers take unsafe behaviors but no accidents occur, then workers’ safety attitudes will increase; if workers take safe behaviors, their safety attitudes will not changes. The formula for calculating the safety attitude is as formula (7).


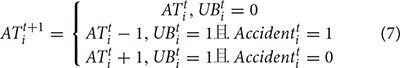


### Experimental Initialization

The environment of construction workers can be divided into operating environment and management environment generally. The strength of the on-site operating environment risk has a strong connection to construction workers’ behavior and decision. Research shows that safety cognition may be related to the work situation of workers and their own safety attitudes. This article considers on-site risk levels. However, as there is no unified classification standard for the classification of environmental risks on construction sites, quantitative analysis is not made in this article. According to the actual construction period of the project, 200 workers are simulated for 200 days. Different values were used to test the influence of these parameters repeatedly, and the results are analyzed and compared to discover objective laws. The settings of other variables in the simulation as shown in Table 1.

**TABLE 1:** Initial value setting of relevant variables.

Variables	Value
Simulation days (D)	200
Number of construction workers (N)	200(20*10)
Actual risk(*AR*)	N(μ, σ^2^)
Initial value of workers’ safety awareness(*SA*)	0.8
Initial value of workers’ safety knowledge(*SK*)	0.8
Initial value of workers’ safety attitude (AT)	0.5
Initial value of risk perception coefficient (p)	U (0.4, 1)
Management norms(*WN*)	U(0.1, 0.9)

Workers with a safety attitude greater than 0.5 indicate that workers have a risk-taking tendency and are more inclined to take unsafe or risk-taking behavior in order to complete the work as soon as possible. The risk perception coefficient is used to reflect the evaluation of construction workers’ perception of risk and whether they can control risk. After repeated tests, it is determined to obey a normal distribution of 0.4 to 1. In this simulation, the management norms follow a uniform distribution within a certain range. When function distribution is not clear, a uniform distribution is often the most appropriate. The values of 0.1 and 0.9 at the two different levels reflect the differences in the management system of managers, indicating that some managers have stricter norms and some are looser, which can more accurately reflect the actual conditions of the construction site. To explore changing law of the number of workers who take unsafe behaviors under different combinations of formal rule awareness (*a*_*i*_) and conformity intention (*c*_*i*_) to conform, the parameter definitions have different values. First, based on the simulation experiment of Byungjoo Choi et al. (Choi and Lee, 2018), the on-site risk level is described as three scenarios. The on-site risk SR is set to 0.45, which means that there is a 45% probability that construction workers are in a dangerous construction site environment. Second, the conformity intention is an important parameter for studying construction workers’ social learning and safety cognition. Seungjun Ahn regards its countdown as the time required for workers to fully recognize the behavior of co-workers and regard it as a formal norm. *c*_*i*_ take 0.05 (Low), 0.4 (Middle), and 0.9 (High). *c*_*i*_ = 0.05 means that construction workers can quickly regard the performance and behavior of co-workers as a code of conduct. A value of 0.9 means that construction workers rely more on the risks they perceive and are less likely to be influenced to believe in their own judgments. Finally, the range of formal rule awareness is from 0.1 to 0.9. It is assumed that construction workers pay more attention to formal rules than internal rules in the formation of risk acceptance. For example, a formal rule awareness of 0.9 means that the relative importance of formal standards and personal standards is 9:1.

## Results and Discussion

**FIGURE 4:**
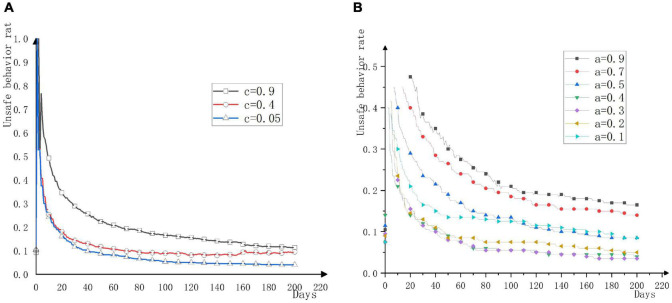
Evolution Trend of unsafe behavior rate under the action of conformity mentality and formal rule awareness. **(A)** Evolution trend of unsafe behavior rate under the action of conformity mentality alone. **(B)** Evolution trend of unsafe behavior rate under the action of formal rule awareness alone.

This model uses Net logo 6.1.1 for simulation. In order to accurately reflect the evolution of workers’ unsafe behavior, each result is run 30 times to achieve an accurate and stable state. In this study, the average unsafe behavior rate or the number of unsafe behaviors in the construction team is used to characterize the influence degree of conformity mentality and formal rule awareness. Figure 4A shows the change trend of unsafe behavior rate when the construction workers’ conformity intention is divided into 0.05, 0.4, and 0.9. The horizontal axis represents the number of simulation days. It can be seen from the figure that in the process of workers’ conformity intention from low to high, the curve of the average unsafe behavior rate of the group gradually rises, and workers tend to adopt unsafe behaviors. Individuals have a high conformity intention to conform to the crowd, and the effect of group conformity is significant. Workers can quickly adapt and adjust their own behavioral norms according to others’ behavioral norms so that the two can reach agreement. This is manifested in workers as that these workers are not so “stubborn,” they easily and quickly regard the co-workers’ behavior as a code of conduct, and the construction team is relatively unstable. Research by Ligia Cremene shows that in the case of complex topologies (scale-free, small world), a high level of conformity seems to be beneficial to dishonest behavior (Cremene and Cremene, 2021). The modern construction environment is becoming more and more complex, and the interaction and connection between the agents are becoming more and more complicated. The traditional simple topology model is gradually not suitable for the building safety simulation model, and the complex topology is more likely to be suitable for modern dynamics. Data from multiple simulation experiments shows that this is closely related to the number of people in the team who initially engage in unsafe behavior. If the initial unsafe number of people in the experiment is large and the conformity mentality level of workers is high, the unsafe behavior rate will increase significantly; if the level of the worker conformity mentality is low, even if the initial number of unsafe behaviors is large, the unsafe behavior rate curve will not show a great upward trend. This is because workers appear to be more “stubborn” and are not easily influenced by others, and the increase in unsafe behavior is not obvious. Therefore, in a construction team with a high level of conformity effect, the composition structure of the construction team and the behavior tendency of the workers must be strictly controlled. Even a small number of unsafe behaviors may have a negative effect on the safety management of the construction team. Managers need to guard against workers with strong conformity mentality, pay attention to the role of psychological adjustment, create a good safety atmosphere through safety education, adjust and guide workers’ psychology, and control the occurrence of unsafe behaviors of the construction team.

Figure 4B shows the changing trend of unsafe behavior rate during the process of construction workers’ formal rule awareness rising from 0.1 to 0.9. Because when the conformity intention of workers is high, workers are more dependent on the behavioral norms of workers, which will cover the influence of formal rule awareness. Therefore, the formal rule awareness should be tested when the level of management norms is high and the conformity mentality of workers is low. As shown in Figure 4B, the influence of formal rule awareness has a critical point, and the average unsafe behavior rate of the group shows a trend of decreasing first and then increasing. When the formal rule awareness is within the range of [0.1, 0.4], the unsafe behavior rate of workers decreases significantly with the improvement of formal rule awareness, continues to increase the value of formal rule awareness, and the unsafe behavior rate of workers increases. The formal rule awareness of construction workers increases within the range of [0.1, 0.4], the closer the internal rules perceived by the workers are to the formal rules. The relationship between workers’ internal rules and formal rules can improve workers’ unsafe behavior, but the gap should not be too large. Thinking that internal rules are particularly important or that formal rules are particularly important are not conducive to workers making correct behavior decisions. In the study of Ahn et al. (2013), the value of *a*_*i*_ was not set too high. In this experiment, the awareness of formal rules was too high, and the difference between the internal rules of the workers and the formal rules was too great, which is not conducive to the construction safety behavior. When workers’ formal rule awareness is low, there are great differences between workers’ perceived internal rules and external rules. According to the basic structure of control theory proposed by Charles S. Carver—feedback loop to reduce differences (Carver and Scheier, 1982), when there are differences between internal rules and formal rules, workers tend to reduce differences to accept unsafe behaviors due to self-regulation. Therefore, from the perspective of formal rule awareness, workers have unsafe behavior in the cognitive process due to the convergence of internal rules and formal rules, which is also verified by the experimental results. Managers should pay attention to the regulatory role of rule awareness in the cognitive process. When the rule awareness is improved within a certain range, the rate of unsafe behavior has been effectively controlled. In view of this, managers should grasp the key factors in the cognitive process and use the self-regulation of construction workers to adapt to formal norms. As the formal rule awareness needs to play a greater role when the management standard is high, while regulating workers’ formal rule awareness, the management must also formulate corresponding rules and regulations, which cannot be lower than the average level of management standard, and otherwise it is difficult to play the corrective role of formal rule awareness.

**FIGURE 5:**
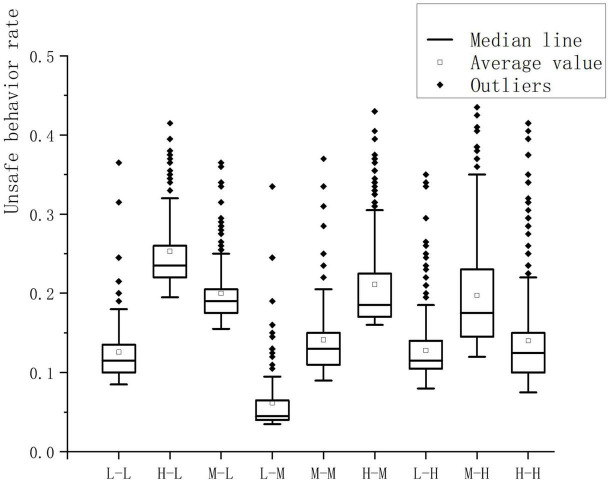
The evolution of unsafe behaviors of workers in the context of different levels of conformity mentality and formal rule awareness.

Figure 5 shows the evolution data of workers’ unsafe behavior under different levels of conformity mentality and formal rule awareness combination scenarios (for example, L-L means low conformity mentality, low formal rule awareness). The outliers in Figure 5 all belong to the right skewed distribution at the upper end of the upper limit. The optimal situation is L-M, the unsafe behavior rate is below 0.1, and the worst situation is H-L, and the unsafe behavior rate reaches 0.25. It can be seen from Figure 5 that in the L-L, L-M, and L-G scenarios, the average unsafe behavior rate is lower, which is a better scenario. At this time, the worker conformity mentality level is at a low level; the average unsafe behavior rate of H-L, M-L, and H-M is higher. It belongs to a poor situation, and the worker’s formal rule awareness is at a low level at this time, which conforms to the law obtained in Figure 4. It can be seen from H-L, H-M, and H-H in Figure 5 that when the conformity mentality of workers is high, the original group unsafe behavior rate should be on the high side. With the improvement of workers’ formal rule awareness, the number of safe behaviors gradually increases. The behavior rate shows a downward trend, which shows that the formal rule awareness has a corrective effect on workers’ unsafe behaviors.

**FIGURE 6:**
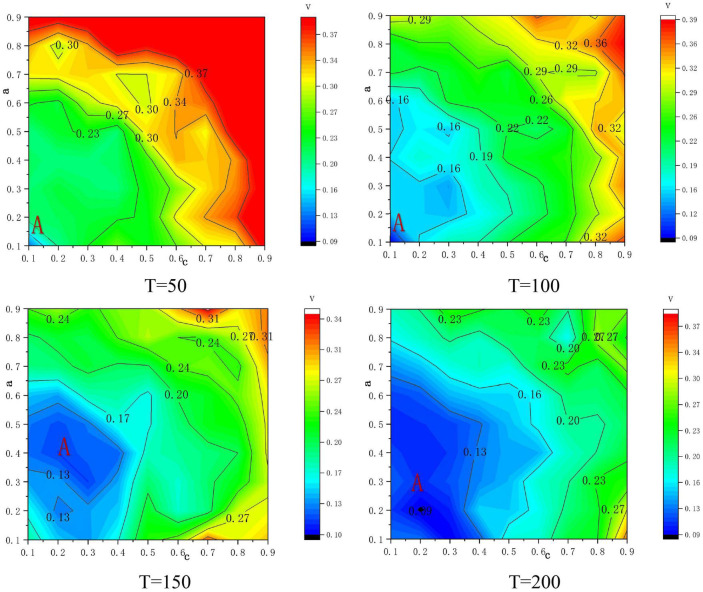
The role of formal rule awareness and conformity mentality in different cycles.

Figure 6 shows the regional map formed by the two-way action of formal rule awareness and conformity mentality of construction workers in different cycles (t = 50, 100, 150, and 200). In the figure, the abscissa axis represents the conformity intention, and the ordinate axis represents the formal rule awareness, which forms the evolution result of the team unsafe behavior rate when the formal rule awareness and conformity mentality act in both directions, and presents a certain hierarchy and regularity.

Not long after the experiment started (T = 50), the formal rule awareness and conformity mentality had not yet taken effect. With the interaction between them, workers’ behavior began to change. As the simulation progresses, the area of area A continues to expand and become more concentrated, which means that in different cycles in the team, the formal rule awareness and the conformity mentality have different effects. In Area A, the rate of unsafe behaviors is low, and workers’ safety awareness remains at a high level. As the formal rule awareness interacts with the conformity mentality, and the value scenarios in Area A are far away, the rate of unsafe behaviors is higher. According to this, in the full-cycle safety production management of building construction, the appropriate degree of interaction between formal rule awareness and conformity mentality can be selected according to the overlapping areas of the area A of different cycles in Figure 6, to achieve the life-cycle safety management of the construction project. When the experiment reaches a steady state, it can be found that as the conformity mentality continues to increase, the unsafe behavior rate presents an upward trend. At this time, the formal rule awareness increases between [0.1, 0.4], and the unsafe behavior rate continues to decrease, so the formal rule awareness can correct unsafe behaviors caused by workers’ cognitive failure, which is consistent with the conclusions in Figure 5.

The above simulation experiments show that when workers cannot accurately perceive risks under certain construction site environmental risks, managers should reasonably control the initial personnel structure of the team according to the characteristics of workers, prevent workers with strong herd mentality, attach importance to workers’ self-regulation, give play to the role of formal rule awareness in correcting deviations in the cognitive process, and create a good safety atmosphere through safety education to regulate and guide workers’ psychology. Adjusting workers’ will to follow the crowd, which can make workers turn to take safety actions and reduce the occurrence of safety accidents. Secondly, in order to achieve higher management objectives, we should adjust the interaction between formal rule awareness and conformity will, make the workers in the state of L = M (low conformity will and medium formal rule awareness), make the workers’ internal rules close to the formal rules and reduce the risk acceptance threshold by formulating reasonable formal rules and safety education, Correct the cognitive failure of workers, so as to take safe behaviors, avoid risks and reduce unsafe behaviors. In the life cycle management of engineering project, the overlapping scope of formal rule awareness and conformity mentality can effectively control the rate of unsafe behavior of the team and ensure the effectiveness of safety management.

## Conclusion

This study uses multi-agent modeling to study the evolution process of construction workers’ unsafe behavior. According to the theories of complex adaptive system, safety cognition and risk perception, the safety cognition rules of unsafe behavior of construction workers are set up, the relationship between safety cognition and unsafe behavior is analyzed, and then a series of simulation experiments are carried out. The model is simulated by different combinations of formal rule awareness and conformity mentality. The simulation results show that: Workers’ unsafe behaviors are inseparable from cognitive failures. Workers’ formal rule awareness and their conformity intention will affect the cognitive process; The higher the level of conformity mentality of workers, the more likely it is to trigger group unsafe behaviors. If the initial number of unsafe behaviors in the team is relatively large, it will aggravate the occurrence of safety accidents; Workers’ formal rule awareness can only play a greater role when management standards are high, and it has a corrective effect on workers’ safety cognition; Under certain construction site environmental risks, the interaction between the formal rule awareness and the conformity mentality within the appropriate range (L-M) is conducive to the realization of the full life cycle management of the engineering project. On the basis of multi-agent modeling, this paper combs the influence relations of various factors in the cognitive process, combines workers’ cognitive process and risk perception, constructs the agent interaction and cognitive model under the two-way effect of formal rule awareness and conformity mentality, promotes the application of safety cognition in regulating construction workers’ safety behavior. Combining computational experiment and cognitive process, the unsafe behavior of workers is studied from the perspective of behavioral evolution. Through simulation, the scene environment of workers’ activities, main agent decision-making and group behavior emergence are reproduced. On this basis, the evolution law is analyzed, which to some extent solves the problems such as difficult modeling in the system and difficult to describe the dynamic interaction process of the main agent, and promotes the process of combining psychological theory with computational simulation method to study practical problems. The findings of this study simulate workers’ cognitive decision-making process by multi-agent modeling, which helps managers understand the motivation and reasons of workers’ behavior from the perspective of individual psychology and consciousness, formulate workers’ safety training system and safety management system, strengthen the management of construction team, and effectively reduce the occurrence of unsafe behavior.

The limitations of this paper can be improved in future research. The description of dynamic changes of management norms or more specific management strategies, worker heterogeneity and learning can be complicated in future research. Through the simulation experiment, the dynamic relationship between unsafe behavior, safety cognition and risk perception of construction workers is improved, which provides a certain direction for the management to formulate the management system from the perspective of safety cognition.

## Data Availability Statement

The original contributions presented in the study are included in the article/supplementary material, further inquiries can be directed to the corresponding author/s.

## Ethics Statement

The study was conducted after approvals were granted from the school of management, Jiangsu University. Written informed consent for participation was not required for this study in accordance with the national legislation and the institutional requirements.

## Author Contributions

XB contributed to conception, modeling, ran the experiment, and writing. ZL and YS contributed to conception and writing. YX contributed to writing and checking. All authors contributed to this article and agreed to the submitted version.

## Conflict of Interest

The authors declare that the research was conducted in the absence of any commercial or financial relationships that could be construed as a potential conflict of interest.

## Publisher’s Note

All claims expressed in this article are solely those of the authors and do not necessarily represent those of their affiliated organizations, or those of the publisher, the editors and the reviewers. Any product that may be evaluated in this article, or claim that may be made by its manufacturer, is not guaranteed or endorsed by the publisher.
